# Effects of folic acid supplementation in pregnant mice on glucose metabolism disorders in male offspring induced by lipopolysaccharide exposure during pregnancy

**DOI:** 10.1038/s41598-023-31690-w

**Published:** 2023-05-17

**Authors:** Wan-Xiao Sun, Yi-Ping Shu, Xin-Yu Yang, Wei Huang, Jing Chen, Ning-Ning Yu, Mei Zhao

**Affiliations:** 1 Department of Basic Nursing, School of Nursing, Anhui Medical University, 81 Meishan Road, Hefei, 230032 Anhui China; 2 Anhui Medical College, Hefei, 230601 Anhui China; 3 Anhui Provincial Hospital, Hefei, 230022 Anhui China; 4 The First Affiliated Hospital of Anhui Medical University, Hefei, 230601 Anhui China

**Keywords:** Endocrinology, Diabetes

## Abstract

The DOHaD theory suggests that adverse environmental factors in early life may lead to the development of metabolic diseases including diabetes and hypertension in adult offspring through epigenetic mechanisms such as DNA methylation. Folic acid (FA) is an important methyl donor in vivo and participates in DNA replication and methylation. The preliminary experimental results of our group demonstrated that lipopolysaccharide (LPS, 50 µg/kg/d) exposure during pregnancy could lead to glucose metabolism disorders in male offspring, but not female offspring; however, the effect of folic acid supplementation on glucose metabolism disorders in male offspring induced by LPS exposure remains unclear. Therefore, in this study, pregnant mice were exposed to LPS on gestational day (GD) 15–17 and were given three doses of FA supplementation (2 mg/kg, 5 mg/kg, or 40 mg/kg) from mating to lactation to explore its effect on glucose metabolism in male offspring and the potential mechanism. This study confirmed that FA supplementation of 5 mg/kg in pregnant mice improved glucose metabolism in LPS-exposed offspring during pregnancy by regulating gene expression.

## Introduction

Developmental Origins of Health and Disease (DOHaD) theory suggests that exposure to nutrition and the environment during pregnancy may put a foetus at higher risk of chronic disease in adulthood^1^. The intrauterine growth period is important for lifelong health, as growth and development of fetal tissues and organ systems occur at a very rapid race, during which either malnutrition or exposure to toxic substances may lead to metabolic disorders in adulthood through epigenetics, increasing the risk of obesity, coronary heart disease, type 2 diabetes (T2D) etc.^2,3^. T2D, also known as non-insulin-dependent or adult diabetes, is an age-related disease that refers to hyperglycaemia caused by impaired insulin secretion and insulin resistance. It accounts for approximately 90% of all diabetes cases in both developing and developed countries^4,5^.

Fu^6^ and Wolfson^7^ showed that maternal lipopolysaccharide (LPS) exposure could cause preterm delivery and embryo resorption in mouse foetuses. In our previous research, exposure to LPS in the middle and late stages of pregnancy caused intrauterine growth restriction, low birth weight and impaired glucose and insulin tolerance in male offspring, but not female offspring^8^.

Folic acid (FA) is essential for maintaining one-carbon unit metabolism in the body and is a universal methyl donor for all biological methylation reactions, thereby participating in cell growth, proliferation, DNA synthesis and methylation^9,10^. Mammalian DNA methylation refers to the addition of methyl groups to cytosine to form 5-methylcytosine catalysed by DNA methyltransferases (DNMTSs)^11,12^. Many diseases are associated with abnormal DNA methylation patterns, such as gene silencing, transcription abnormalities, and phenotypic changes caused by methylation of tumor suppressor gene promoters, which is a common observation in cancer^11^. The DNA methylation process can be reversed by nutrition, the environment and other factors^13^. FA can regulate gene DNA methylation levels, optimize DNA repair ability, and ensure DNA authenticity and integrity^14–16^. The US Preventive Services Task Force (USPSTF) recommends that all women who are planning or capable of pregnancy take a daily supplement containing 0.4–0.8 mg (400–800 µg) of FA to reduce the rate of neural tube defects (NTDs)^17^. In high-risk groups, FA supplements of 4–5 mg per day are recommended to reduce the occurrence or recurrence of NTDs in infants^18^.

However, the effect of FA supplementation on the susceptibility of offspring to glucose metabolism disorders caused by LPS exposure during pregnancy remains to be studied. Therefore, the present study used CD-1 mice to explore the effect of FA supplementation on glucose metabolism in offspring exposed to LPS during pregnancy and its potential mechanism.

## Materials and methods

### Folic acid

Based on the standard AIN-93G diet feed^19^, three groups of FA diet (cat. nos. LAD-3001G-FA2, LAD-3001G-F5 and LAD-3001G-FA40; Trophic Animal Feed High-Tech Co., Ltd.) were designed: i) the FA2 diet (consisting of 2 mg/kg FA, which is the basic requirement for rodents); ii) the FA5 diet, consisting of 5 mg/kg FA; and iii) the FA40 diet consisting of 40 mg/kg FA (Table 1). It is generally accepted that 2 mg/kg FA is the basic requirement for rodents^19^. The doses of FA2, FA5 and FA40 are equivalent to 400 μg, 800–1000 μg and 4 mg of FA per day in pregnant women, respectively^20^.

**Table 1:** Folic acid feed composition.

Ingredient	FA2	FA5	FA40
Carbohydrate (%)	64.3	64.3	64.3
protein (%)	17.8	17.8	17.8
fat (%)	7	7	7
Vitamin content			
Folic acid (mg/kg)	2	5	40
Vitamin A (IU/g)	4	4	4
Vitamin D (IU/g)	1	1	1
Vitamin E (IU/g)	0.075	0.075	0.075
Vitamin K (mg/kg)	0.9	0.9	0.9
Vitamin B1 (mg/kg)	5	5	5
Vitamin B2 (mg/kg)	6	6	6
Vitamin B3 (mg/kg)	30	30	30
Vitamin B5 (mg/kg)	15	15	15
Vitamin B6 (mg/kg)	6	6	6
Vitamin B4 (mg/kg)	1000	1000	1000
Vitamin H (mg/kg)	0.2	0.2	0.2
Mineral content			
Calcium (mg/kg)	5000	5000	5000
phosphorus (mg/kg)	3000	3000	3000
potassium (mg/kg)	3600	3600	3600
sodium (mg/kg)	1039	1039	1039
magnesium (mg/kg)	513	513	513
iron (mg/kg)	45	45	45
zinc (mg/kg)	38	38	38
fierce (mg/kg)	10	10	10
copper (mg/kg)	6	6	6
iodine (mg/kg)	0.2	0.2	0.2
chromium (mg/kg)	1	1	1
Inorganic sulfur (mg/kg)	300	300	300
chlorine (mg/kg)	1631	1631	1631
Amino acid content			
alanine (g/kg)	4.6	4.6	4.6
arginine (g/kg)	6.4	6.4	6.4
aspartic acid (g/kg)	12.2	12.2	12.2
glutamic acid (g/kg)	36.3	36.3	36.3
glycine (g/kg)	3.2	3.2	3.2
lysine (g/kg)	13	13	13
methionine (g/kg)	4.6	4.6	4.6
cystine (g/kg)	3.7	3.7	3.7
tryptophan (g/kg)	2.1	2.1	2.1
proline (g/kg)	20.5	20.5	20.5
serine (g/kg)	9.7	9.7	9.7
histidine (g/kg)	4.6	4.6	4.6
leucine (g/kg)	15.4	15.4	15.4
isoleucine (g/kg)	8.5	8.5	8.5
phenylalanine (g/kg)	8.8	8.8	8.8
tyrosine (g/kg)	9.3	9.3	9.3
threonine (g/kg)	6.7	6.7	6.7
valine (g/kg)	10	10	10

### Animals and treatments

A total of 60 female (weight, 28–30 g) and 30 male (weight, 30–32 g) CD-1 mice were purchased from Beijing Vital River Laboratory Animal Technology Co., Ltd. at 8 weeks of age. All procedures performed on animals were in accordance with the national standards for animal experiments^21^ and the ARRIVE guidelines. The present study was approved by the Ethics Committee of Anhui Medical University (approval no. LLSC20150350; Hefei, China). A total of two mice were housed per cage under a 12-h dark/light cycle in a controlled environment (temperature, 20–24 °C; relative humidity, 50–55%) and left to acclimatize for 2 weeks. After adaptive feeding, mating was performed. The day of successful mating was considered gestational day 0 (GD0).

Sixty female mice were randomly divided into 6 group, namely, the low-dose FA group (FA2) + normal saline (NS), medium-dose FA group (FA5) + normal saline (NS), high-dose FA group (FA40) + normal saline (NS), low-dose FA group (FA2) + LPS, medium-dose FA group (FA5) + LPS, and high-dose FA group (FA40) + LPS. The offspring were FA2-NS, FA5-NS, FA40-NS, FA2-LPS, FA5-LPS, and FA40-LPS. NS and LPS were injected intraperitoneally on GD15-17 at a dose of 50 µg/kg/d. FA2, FA5 and FA40 were diets containing different doses of FA (2 mg/kg, 5 mg/kg and 40 mg/kg), and the feeding time was from the adaptive feeding period to the end of lactation. All pregnant mice delivered naturally. The birth date was postnatal day 1 (PD1). The offspring were weaned 21 days after birth (PD21), after which they were all given the FA2 diet. Glucose tolerance tests (GTT) were performed at PD60, PD90 and PD120, and insulin tolerance tests (ITT) were performed at PD120. After fasting for 12 h, the serum was taken to detect serum insulin, triglyceride (TG) and total cholesterol (TC) contents at PD120. The protein expression of phosphatidylinositol 3 kinase (PI3K), phosphorylated phosphatidylinositol 3 kinase (p-PI3K), protein kinase B (AKT), phosphorylated protein kinase B (p-Akt), glycogen synthase kinase3 (GSK3β) and phosphorylated glycogen synthase kinase3β (p-GSK3β) and the mRNA expression of *dnmt1*, *dnmt3a*, *dnmt3b*, peroxisome proliferator-actived receptor γ (*pparγ)*, insulin like growth factor 2 (*igf2*) and Polyethylene glycol 3 (*peg3)* were detected in liver tissues.

### Fasting blood glucose, glucose tolerance test and insulin tolerance test

#### Fasting blood glucose (FBG)

FBG was measured once a week after adulthood (on PD60, PD77, PD84, PD90, PD98, PD105, PD112, and PD120). Eight male offspring were randomly selected from each group and fasted for 12 h, and the FBG level of mice was measured by tail bleeding using a Roche glucometer (cat. no. 06993788001; Roche, Inc.).

#### GTT and ITT

At PD60, PD90 and PD120, 8 male offspring in each group were randomly selected to fast for 4 h and injected with glucose (cat. no. DB02-2.0701; China Otsuka Pharmaceutical Co., Ltd.) at 2.0 g/kg body weight intraperitoneally. At PD120, 8 male offspring in each group were randomly selected to fast for 4 h and injected with insulin (cat. no. 9004–10-8; Wanbang Biopharmaceuticals Co., Ltd.) at 0.75 U/kg body weight intraperitoneally. FBG (0 min) and blood glucose levels at 15 min, 30 min, 60 min and 120 min after injection of glucose or insulin were measured.

### Western blot (WB)

At PD120, the livers of mice were collected and homogenized using 0.4 ml of lysis buffer containing 50 mM Tris–HCl, 150 mM NaCl, 1 mM EDTA, 1% Triton X-100, 1% sodium deoxycholate and 0.1% SDS, supplemented with 1% protease inhibitors, 1% PMSF and 2% phosphatase inhibitor. After centrifugation at 15,000 × g for 15 min at 4 °C, protein concentrations were measured using the BCA assay. The samples were then boiled for 10 min at 100 °C, after which equal amounts of protein (30 μg) were separated by SDS‒PAGE (12.5% or 15%) and transferred onto PVDF membranes. After blocking in blocking buffer (5% skimmed milk powder) at room temperature for 1.5 h, blots were incubated overnight with antibodies against p-PI3K (1:1,000; cat. no. 4228 T; Cell Signaling Technology, Inc.), PI3K (1:1,000; cat. no. 4257S; Cell Signaling Technology, Inc.), p-AKT (1:1,000; cat. no. 4060S; Cell Signaling Technology, Inc.), AKT (1:2,000; cat. no. 4691S; Cell Signaling Technology, Inc.), p-GSK3β (1:1,000; cat. no. 9323S; Cell Signaling Technology, Inc.), GSK3β (1:1,000; cat. no. 9315; Cell Signaling Technology, Inc.) and GAPDH (1:1,000; cat. no. 365062; Sant Cruz, Inc.) at 4 °C. After washing three times, the membrane was probed with HRP-conjugated goat anti-rabbit IgG antibodies (1:10,000; cat. no. sc-2005; Santa Cruz Biotechnology, Inc.) for 1 h at room temperature. The blots were subsequently detected using an ECL detection kit (Pierce; Thermo Fisher Scientific, Inc.) and quantified using a ChemiDoc Imaging system (version, 2.3.0.07; Bio-Rad, Inc.).

### Real-time PCR

Total RNA was extracted from the liver using TRIzol reagent (cat. no. 15596026; Thermo Scientific, Inc). Next, 4.0 μL of total RNA sample was reverse transcribed into cDNA through denaturation, reverse transcription, incubation and other steps, and 1.0 μL of cDNA was amplified by PCR (version, Light Cycle®480; Roche, Inc.). Gene primer sequences are shown in Table 2.

**Table 2:** Gene primer sequences.

Genes	Sequences 5’-3’
*dnmt1*	Forward:GGACAGTGACACCCTTTCAGT
	Reverse: GAGTTCCCCTCTTCCGACTC
*dnmt3a*	Forward: GCTCGGACCCCGCAACT
	Reverse: GCCAGACCTTGGAAACCTCA
*dnmt3b*	Forward: GCTGGCACCCTCTTCTTCAT
	Reverse: GCTGGCACCCTCTTCTTCAT
*pparγ*	Forward:GACGCGGAAGAAGAGACCTG
	Reverse: GTGTGACTTCTCCTCAGCCC
*igf2*	Forward: CACTTCTCCTACGGTGTCCC
	Reverse: GGCAATGCCCAGTCGTTTTC
*peg3*	Forward: CTGTCATCGATCCCGACTGG
	Reverse: CACCTAGCTGTTGGAGGACC
*18 s*	Forward: GTAACCCGTTGAACCCCATT
	Reverse: CCATCCAATCGGTAGTAGCG

### Biochemical analysis

Serum samples from all the mice were collected through centrifugation at 12,000 × g for 15 min at 4 °C and stored at -80 °C. The serum levels of TG and TC were measured using the glycerol phosphate hydrogenase method (TG kit, cat. no. A110-1–1; Nanjing Jiancheng Bioengineering Institute, Inc.) and cholesterol oxidase method (TC kit, cat. no. A111-2–1; Nanjing Jiancheng Bioengineering Institute, Inc.) separately by an automatic biochemical analyser (cat. no. CS-T300; Dirui Industrial Co., Ltd.).

### Enzyme-linked immunosorbent assay (ELISA)

Serum insulin levels were detected using the Mouse Insulin ELISA Kit (cat. no. CSB-E05071m; Cusabio Biotech Co., Ltd.) according to the guidelines.

### Statistical analysis

SPSS 23.0 (IBM Corp.) and GraphPad Prism 9 (GraphPad Software, Inc.) software were used to analyse the data, which are presented as the mean ± SD. Body weight, FBG, GTT and ITT were analysed using mixed ANOVA followed by Tukey’s test. In mixed ANOVA, repeated measurements at time points were an within-subjects factor, while FA diet or LPS treatment was an between-subjects factor. Unpaired two-tailed Student’s t test was used to analyse the liver weight, liver/body weight ratio, TG, TC, the area under the glucose curve (AUC), fasting insulin levels, and WB and mRNA expression data of the two offspring groups when pregnant mice were given the same dose of FA supplementation. One-way ANOVA and the SNK test were used to analyse the liver weight, liver/body weight ratio, TG, TC, AUC, fasting insulin levels, and WB and mRNA expression data of the three groups (FA2-LPS, FA5-LPS, and FA40-LPS) of offspring, and the same method was used to compare the FA2-NS, FA5-NS, and FA40-NS groups. *P *< 0.05 was considered to indicate a statistically significant difference.

### Ethical approval

This protocol has received ethics approval from the Ethics Committee of AnhuiMedical University and the ethical approval number is LLSC20150350.

## Results

### Effects of FA supplementation in pregnant mice on offspring weight

**Figure 1:**
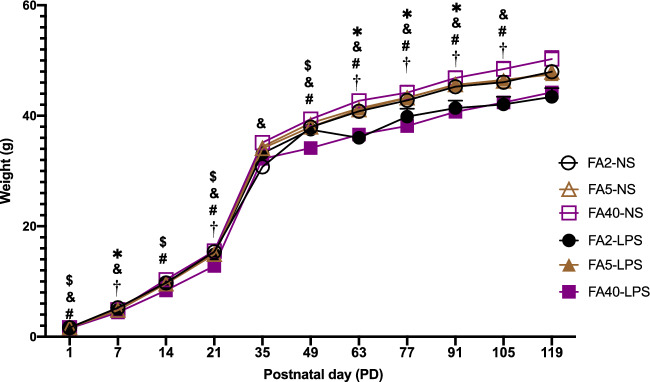
The offspring's body weight (n = 24 per group ). All data were presented as mean ± SD. Mixed ANOVA: † *P *< 0.05, FA2-LPS vs FA2-NS, # *P *< 0.05, FA40-LPS vs FA40-NS. Mixed ANOVA followed by Tukey’s test: * *P *< 0.05, FA2-LPS vs FA5-LPS, $ *P *< 0.05, FA2-LPS vs FA5-LPS, & *P *< 0.05, FA5-LPS vs FA40-LPS. The birth date is postnatal day 1 (PD1). FA2-NS, FA5-NS, FA40-NS, offspring of pregnant mice not exposed to LPS and supplemented with FA at a dose of 2 mg/kg, 5 mg/kg and 40 mg/kg respectively. FA2-LPS, FA5-LPS, FA40-LPS, offspring of pregnant mice exposed to LPS and supplemented with FA at a dose of 2 mg/kg, 5 mg/kg and 40 mg/kg respectively.

When pregnant mice were fed the same dose of FA in the diet, the weight of offspring in the FA2-LPS group was lower than that in the FA2-NS group (except at PD1, PD14, PD35, PD49 and PD119) (*P *< 0.05). The offspring body weight of the FA40-LPS group was lower than that of the FA40-NS group (except at PD7, PD35 and PD119) (*P *< 0.05). There was no significant difference between the FA5-LPS and FA5-NS groups (*P *> 0.05) (Fig. 1).

When pregnant mice were not exposed to LPS in the late stage of pregnancy (injected intraperitoneally with NS on GD15-17), there was no significant difference in body weight at any time point among the FA2-NS, FA5-NS and FA40-NS groups (*P *> 0.05) (Fig. 1).

When pregnant mice were exposed to LPS at the late stage of pregnancy, the offspring body weight of the FA2-LPS group was lower than that of the FA5-LPS group on PD7 and PD63-91, and the offspring body weight of the FA40-LPS group was lower than that of the FA5-LPS group (except at PD14 and PD119). The offspring body weight of the FA40-LPS group at PD1, PD14, PD21 and PD49 was lower than that of the FA2-LPS group (*P *< 0.05) (Fig. 1).

### Effects of FA supplementation in pregnant mice on the FBG of offspring

**Figure 2:**
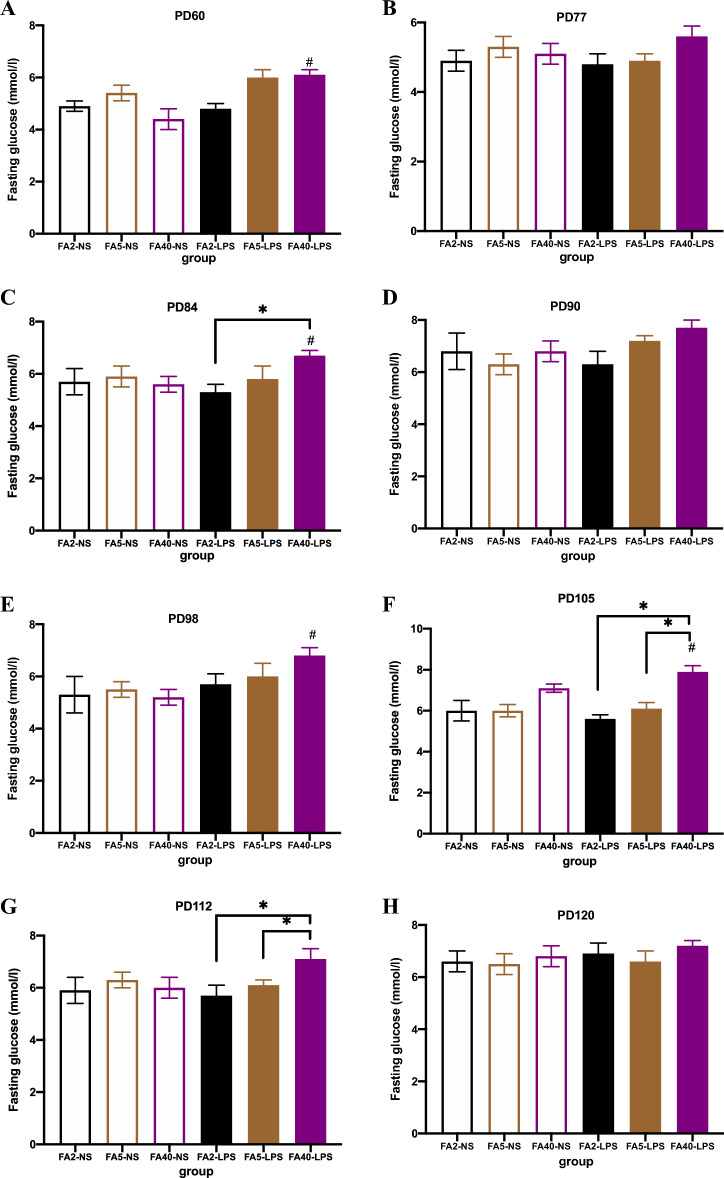
Fasting blood glucose in offspring (n = 8 per group). Fasting blood glucose levels of offspring at (**A**) PD60, (**B**) PD77, (**C**) PD84, (**D**) PD90, (**E**) PD98, (**F**) PD105, (**G**) PD112 and (**H**) PD120. All data were presented as mean ± SD. Mixed ANOVA: # *P *< 0.05, FA40-LPS vs FA40-NS. Mixed ANOVA followed by Tukey’s test: * *P *< 0.05, FA2-LPS vs FA5-LPS, FA2-LPS vs FA40-LPS, FA5-LPS vs FA40-LPS. PD, postnatal day, the birth date is PD1. FA2-NS, FA5-NS, FA40-NS, offspring of pregnant mice not exposed to LPS and supplemented with FA at a dose of 2 mg/kg, 5 mg/kg and 40 mg/kg respectively. FA2-LPS, FA5-LPS, FA40-LPS, offspring of pregnant mice exposed to LPS and supplemented with FA at a dose of 2 mg/kg, 5 mg/kg and 40 mg/kg respectively.

When pregnant mice were fed the same dose of FA in the diet, the FBG of the offspring of the FA40-LPS group was higher than that of the FA40-NS group at PD60, PD84, PD98 and PD105 (*P *< 0.05). There was no significant difference in the FBG levels between the FA2-LPS and FA2-NS groups or between the FA5-LPS and FA5-NS groups (*P *> 0.05) (Fig. 2).

When pregnant mice were not exposed to LPS, there was no significant difference in FBG levels at any time point among the FA2-NS, FA5-NS and FA40-NS groups (*P *> 0.05) (Fig. 2).

When pregnant mice were exposed to LPS at the late stage of pregnancy, the FBG level of male offspring in the FA5-LPS group was lower than that in the FA40-LPS group at PD105 and PD112, and the FBG level of male offspring in the FA2-LPS group was lower than that in the FA40-LPS group at PD84, PD105 and PD112 (*P *< 0.05) (Fig. 2).

### Effects of FA supplementation in pregnant mice on the GTT of offspring

**Figure 3:**
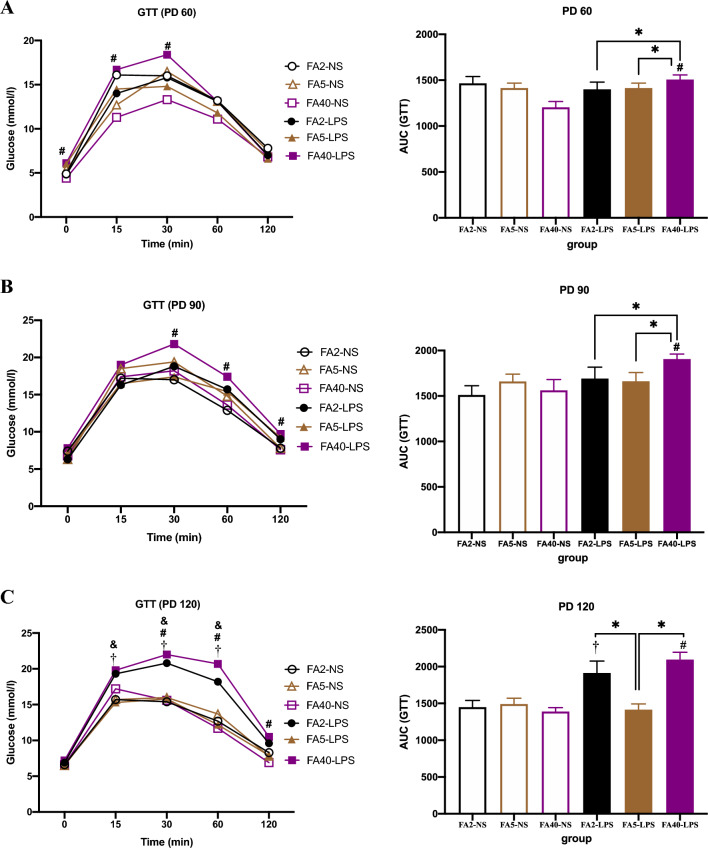
Glucose tolerance test in offspring (n = 8 per group). GTT and AUC of offspring at (**A**) PD60, (**B**) PD90 and (**C**) PD120. All data were presented as mean ± SD. Mixed ANOVA in GTT and Student’s t-test in AUC: † *P *< 0.05, FA2-LPS vs FA2-NS. # *P *< 0.05, FA40-LPS vs FA40-NS. Mixed ANOVA followed by Tukey’s test in GTT : & *P *< 0.05, FA5-LPS vs FA40-LPS. One-way ANOVA and SNK test in AUC:* *P *< 0.05, FA2-LPS vs FA5-LPS, FA2-LPS vs FA40-LPS, FA5-LPS vs FA40-LPS. PD, postnatal day, the birth date is PD1. GTT, glucose tolerance test. AUC, area under curve. FA2-NS, FA5-NS, FA40-NS, offspring of pregnant mice not exposed to LPS and supplemented with FA at a dose of 2 mg/kg, 5 mg/kg and 40 mg/kg respectively. FA2-LPS, FA5-LPS, FA40-LPS, offspring of pregnant mice exposed to LPS and supplemented with FA at a dose of 2 mg/kg, 5 mg/kg and 40 mg/kg respectively.

When pregnant mice were fed the same dose of FA in the diet, AUC in the FA2-LPS group was higher than that in the FA2-NS group at PD120 (*P *< 0.05). The FA40-LPS group had higher blood glucose and AUC values than those of the Ctrl group (40 mg) at PD60, PD90 and PD120 (*P *< 0.05). There were no significant differences in the blood glucose and AUC values between the FA5-LPS and FA5-NS groups (*P *> 0.05) (Fig. 3).

When pregnant mice were not exposed to LPS, there was no significant difference in the blood glucose levels and AUC among the FA2-NS, FA5-NS and FA40-NS groups at any time point (*P *> 0.05) (Fig. 3).

When the pregnant mice were exposed to LPS at the late stage of pregnancy, the AUC in the FA2-LPS and FA5-LPS groups was lower than that in the FA40-LPS group at PD60 and PD90 (*P *< 0.05). At PD120, the AUC in the FA5-LPS group was lower than that in the FA2-LPS and FA40-LPS groups, and the blood glucose in the FA5-LPS group was lower than that in the FA40-LPS group at 15 min, 30 min and 60 min (*P *< 0.05) (Fig. 3).

### Effects of FA supplementation in pregnant mice on the ITT of offspring

**Figure 4:**
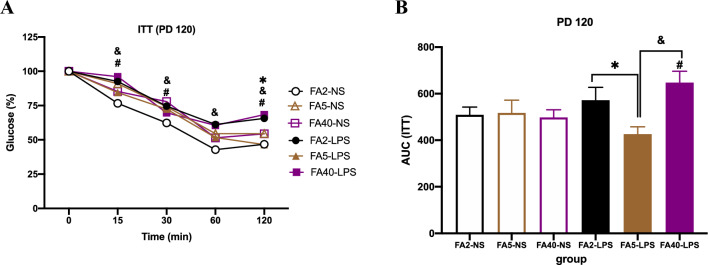
Insulin tolerance test of offspring (n = 8 per group). (**A**) ITT and (**B**) AUC of offspring at PD120. All data were presented as mean ± SD. Mixed ANOVA in GTT and Student’s t-test in AUC: # *P *< 0.05, FA40-LPS vs FA40-NS. Mixed ANOVA followed by Tukey’s test in GTT and one-way ANOVA and SNK test in AUC: * *P *< 0.05, FA2-LPS vs FA5-LPS. & *P *< 0.05, FA5-LPS vs FA40-LPS. PD, postnatal day, the birth date is PD1. GTT, glucose tolerance test. AUC, area under curve. FA2-NS, FA5-NS, FA40-NS, offspring of pregnant mice not exposed to LPS and supplemented with FA at a dose of 2 mg/kg, 5 mg/kg and 40 mg/kg respectively. FA2-LPS, FA5-LPS, FA40-LPS, offspring of pregnant mice exposed to LPS and supplemented with FA at a dose of 2 mg/kg, 5 mg/kg and 40 mg/kg respectively.

When pregnant mice were fed the same dose of FA in the diet, the blood glucose and AUC values in the FA40-LPS group were higher than those in the FA40-NS group (*P *< 0.05). Compared with those of the FA2-NS and FA5-NS groups, there was no significant difference in the blood glucose and AUC values between the FA2-NS and FA2-LPS groups or between the FA5-NS and FA5-LPS groups (*P *> 0.05) (Fig. 4).

When pregnant mice were not exposed to LPS, there was no significant difference in blood glucose at any time point among the FA2-NS, FA5-NS and FA40-NS groups (*P *> 0.05) (Fig. 4).

When pregnant mice were exposed to LPS at the late stage of pregnancy, the AUC in the FA5-LPS group was lower than that in the FA2-LPS group and the FA40-LPS group (*P *< 0.05). The blood glucose levels in the FA5-LPS group were lower than those in the FA40-LPS group at 15 min, 30 min and 120 min (*P *< 0.05) (Fig. 4).

### Effects of FA supplementation in pregnant mice on the fasting serum insulin levels of offspring

**Figure 5:**
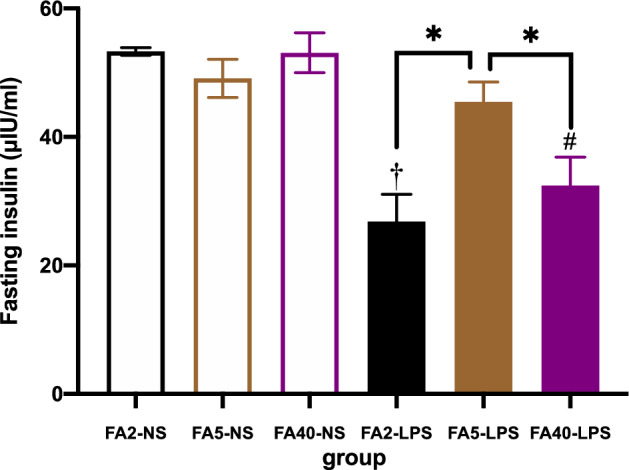
Fasting serum insulin of offspring (n = 8 per group). All data were presented as mean ± SD. Student’s t-test: † *P *< 0.05, FA2-LPS vs FA2-NS. # *P *< 0.05, FA40-LPS vs FA40-NS. One-way ANOVA and SNK test: * *P *< 0.05, FA2-LPS vs FA5-LPS, FA2-LPS vs FA40-LPS, FA5-LPS vs FA40-LPS. FA2-NS, FA5-NS, FA40-NS, offspring of pregnant mice not exposed to LPS and supplemented with FA at a dose of 2 mg/kg, 5 mg/kg and 40 mg/kg respectively. FA2-LPS, FA5-LPS, FA40-LPS, offspring of pregnant mice exposed to LPS and supplemented with FA at a dose of 2 mg/kg, 5 mg/kg and 40 mg/kg respectively.

When pregnant mice were fed the same dose of FA in the diet, the fasting serum insulin levels in the FA2-LPS and FA40-LPS groups were lower than those in the FA2-NS and FA40-NS groups, respectively (*P *< 0.05). There was no significant difference in fasting serum insulin levels between the FA5-LPS and FA5-NS groups (*P *> 0.05) (Fig. 5).

When pregnant mice were not exposed to LPS, there was no significant difference in the fasting serum insulin levels among the FA2-NS, FA5-NS and FA40-NS groups (*P *> 0.05) (Fig. 5).

When pregnant mice were exposed to LPS in the late stage of pregnancy, the fasting serum insulin level in the FA5-LPS group was higher than that in the FA2-LPS group and the FA40-LPS group (*P *< 0.05) (Fig. 5).

### Effects of FA supplementation in pregnant mice on the insulin signalling pathway of offspring

**Figure 6:**
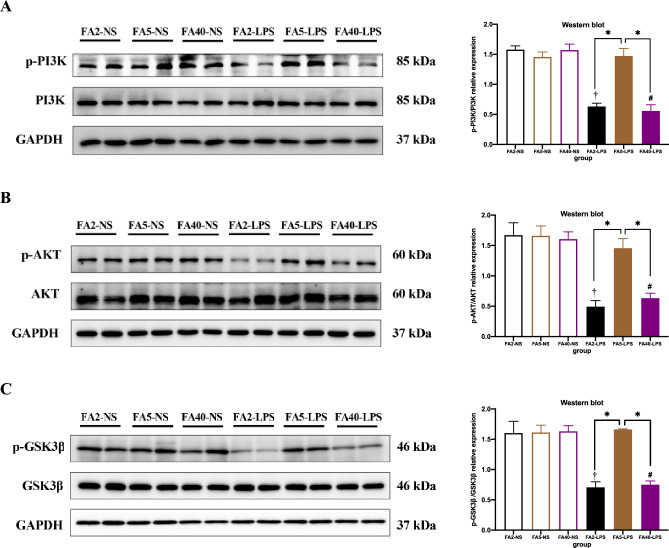
Insulin signaling pathway in offspring (n = 8 per group). Representative western blots and corresponding quantification of the levels of (**A**) PI3K and p-PI3K, (**B**) AKT and p-AKT, (**C**) GSK3β and p-GSK3β. All data were expressed as mean ± SD. Student’s t-test: † *P *< 0.05, FA2-LPS vs FA2-NS. # *P *< 0.05, FA40-LPS vs FA40-NS. One-way ANOVA and SNK test: * *P *< 0.05, FA2-LPS vs FA5-LPS, FA2-LPS vs FA40-LPS, FA5-LPS vs FA40-LPS. p-, phosphorylated. FA2-NS, FA5-NS, FA40-NS, offspring of pregnant mice not exposed to LPS and supplemented with FA at a dose of 2 mg/kg, 5 mg/kg and 40 mg/kg respectively. FA2-LPS, FA5-LPS, FA40-LPS, offspring of pregnant mice exposed to LPS and supplemented with FA at a dose of 2 mg/kg, 5 mg/kg and 40 mg/kg respectively. All blots were cut prior to hybridisation with antibodies.

When pregnant mice were fed the same dose of FA in the diet, the protein expression of p-PI3K, p-Akt and p-GSK3β in the liver in the FA2-LPS and FA40-LPS groups was lower than that in the FA2-NS and FA5-NS groups (*P *< 0.05), although the protein expression of PI3K, AKT and GSK3β was not significantly different. The protein expression of p-PI3K, PI3K, p-Akt, AKT, p-GSK3β and GSK3β in the liver in the FA5-LPS group was not significantly different from that in the FA5-NS group (*P *> 0.05) (Fig. 6).

When pregnant mice were not exposed to LPS, there was no significant difference in the protein expression of p-PI3K, PI3K, p-Akt, AKT, p-GSK3β and GSK3β among the FA2-NS, FA5-NS and FA40-NS groups (*P *> 0.05) (Fig. 6).

When pregnant mice were exposed to LPS in late pregnancy, the protein expression of p-PI3K, p-Akt and p-GSK3β in the liver in the FA5-LPS group was higher than that in the FA2-LPS and FA40-LPS groups (*P *< 0.05). There was no significant difference in the protein expression of AKT, p-GSK3β and GSK3β among the three groups (*P *> 0.05) (Fig. 6, Supplymentary Fig. 6).

### Effects of FA supplementation in pregnant mice on serum TG, TC, liver weight and liver/body weight ratio of offspring

**Figure 7:**
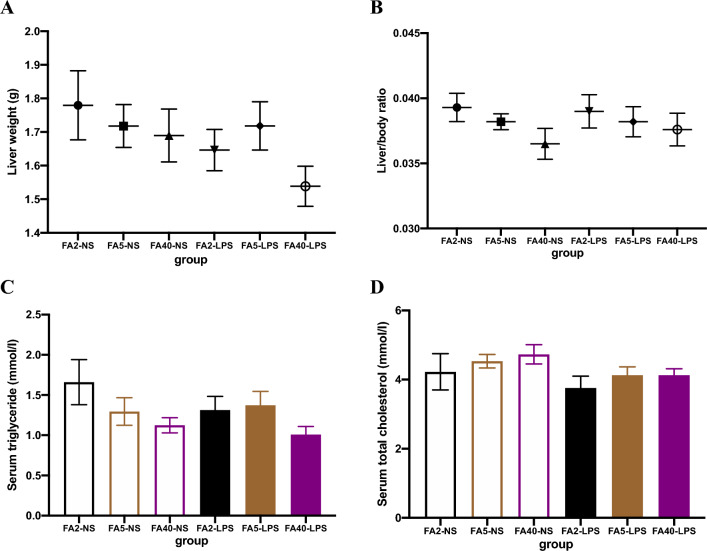
Liver weight, liver/body ratio, serum triglyceride and total cholesterol of offspring (n = 8 per group). (**A**) liver weight. (**B**) Liver weight factor (liver weight/body weight). (**C**) Serum triglyceride levels. (**D**) Serum total cholesterol level. All data were presented as mean ± SD. FA2-NS, FA5-NS, FA40-NS, offspring of pregnant mice not exposed to LPS and supplemented with FA at a dose of 2 mg/kg, 5 mg/kg and 40 mg/kg respectively. FA2-LPS, FA5-LPS, FA40-LPS, offspring of pregnant mice exposed to LPS and supplemented with FA at a dose of 2 mg/kg, 5 mg/kg and 40 mg/kg respectively.

When pregnant mice were fed the same dose of FA, there were no significant differences in the liver weight, liver/body weight ratio, or TG and TC levels between the FA2-LPS and FA2-NS groups, the FA5-LPS and FA5-NS groups, or the FA40-LPS and FA40-NS groups (*P *> 0.05) (Fig. 7).

Whether or not the pregnant mice were exposed to LPS in late pregnancy, there were no statistically significant differences in the liver weight, liver/body weight ratio, or the TG and TC levels among the FA2-NS, FA5-NS and FA40-NS groups or among the FA2-LPS, FA5-LPS and FA40-LPS groups (*P *> 0.05) (Fig. 7).

### Effects of FA supplementation in pregnant mice on liver mRNA expression in offspring

**Figure 8:**
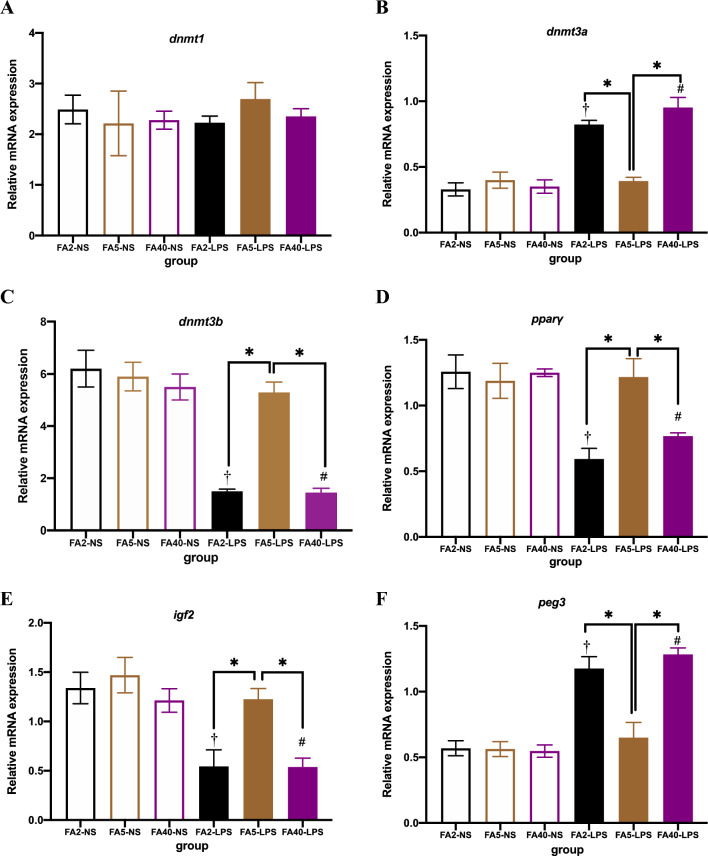
mRNA levels in offspring (n = 8 per group). mRNA levels of (**A**) *dnmt1*, (**B**) *dnmt3a*, (**C**) *dnmt3b*, (**D**) *pparγ*, (**E**) *igf*2, (**F**) *peg3*. All data were presented as mean ± SD. Student’s t-test: † *P *< 0.05, FA2-LPS vs FA2-NS. # *P *< 0.05, FA40-LPS vs FA40-NS. One-way ANOVA and SNK test: * *P *< 0.05, FA2-LPS vs FA5-LPS, FA2-LPS vs FA40-LPS, FA5-LPSvs FA40-LPS. FA2-NS, FA5-NS, FA40-NS, offspring of pregnant mice not exposed to LPS and supplemented with FA at a dose of 2 mg/kg, 5 mg/kg and 40 mg/kg respectively. FA2-LPS, FA5-LPS, FA40-LPS, offspring of pregnant mice exposed to LPS and supplemented with FA at a dose of 2 mg/kg, 5 mg/kg and 40 mg/kg respectively.

When pregnant mice were fed the same dose of FA in the diet, the mRNA levels of *dnmt3b*, *pparγ* and *igf2* in the FA2-LPS and FA40-LPS groups were lower than those in the FA2-NS and FA40-NS groups, respectively (*P *< 0.05), and the mRNA levels of *dnmt3a* and *peg3* were higher than those in the FA2-NS and FA40-NS groups, respectively (*P *< 0.05). There was no significant difference in the mRNA levels between the FA5-LPS and FA5-NS groups (*P *> 0.05). There was no significant difference in the *dnmt1* levels between any of the two groups at the same supplemental dose of FA (Fig. 8).

When pregnant mice were not exposed to LPS, there was no significant difference in the mRNA levels of each gene among the FA2-NS, FA5-NS and FA40-NS groups (*P *> 0.05) (Fig. 8).

When exposed to LPS in late pregnancy, the mRNA levels of *dnmt3b*, *pparγ* and *igf2* in the FA5-LPS group were higher than those in the FA2-LPS and FA40-LPS groups, and the mRNA levels of *dnmt3a* and *peg3* were lower than those in the FA2-LPS and FA40-LPS groups (*P *< 0.05). There was no significant difference in the *dnmt1* levels among the three groups (Fig. 8).

## Discussion

In the early stage of life, especially during intrauterine development, adverse environmental factors can change the genotype of cells through epigenetic mechanisms such as DNA methylation and can ultimately affect the occurrence and development of related diseases in adulthood (such as diabetes, hypertension, coronary heart disease and metabolic syndrome), which is called the DOHaD theory^1,2^. Our preliminary experimental results demonstrated that LPS exposure during pregnancy can lead to glucose metabolism disorder in male offspring (PD60)^8^. The correct establishment and maintenance of DNA methylation patterns are crucial for foetal survival and growth, and FA is an important methyl donor in vivo and participates in DNA replication and DNA methylation^9^. Therefore, based on a previous experiment, this study further investigated the effects of FA supplementation at different doses on glucose metabolism disorder in LPS-exposed male offspring.

When pregnant mice were not exposed to LPS (pregnant mice were injected with LPS on GD15-17), FA supplementation at different doses (2 mg/kg, 5 mg/kg, and 40 mg/kg) had no significant effect on the body weight and glucose metabolism of their male offspring. This is consistent with the study of Huang et al.^20^, which pointed out that when the offspring were fed the AIN-93G control diet, supplementation with FA at different doses had no significant effect on the body weight, FBG, insulin resistance index, TGs, TC and other lipids of the offspring.

When pregnant mice were exposed to LPS in late pregnancy, supplementation with FA at 2 mg/kg and 40 mg/kg resulted in lower birth weight, reduced fasting serum insulin levels, impaired PI3K/AKT insulin signalling pathways and impaired glucose homeostasis in their offspring. Glucose homeostasis is the result of the tight maintenance of blood glucose levels in response to internal changes or external events such as feeding, fasting, stress, or exercise^22^. Insulin is a peptide hormone secreted by pancreatic β cells that can bind to insulin receptors and activate PI3K by phosphorylating it ^23,24^. P-PI3K phosphorylates phosphatidylinositol 4,5-diphosphate to phosphatidylinositol 3,4,5-triphosphate and further phosphorylates AKT. AKT activation can inhibit GSK3β-induced glycogen storage and regulate systemic glucose homeostasis^23,24^. An Indian birth cohort study showed that higher maternal FA concentrations were associated with higher insulin resistance in children at 9.5 and 13.5 years of age^25^. Our results suggested that maternal high FA intake accelerates the development of obesity in male offspring^26^. Zetstra-van der Woude, P. A. suggested that supplementation with high-dose FA during pregnancy might increase the risk of childhood asthma^27^.

When pregnant mice were exposed to LPS in late pregnancy, compared with 2 mg/kg and 40 mg/kg FA supplementation in pregnant mice, 5 mg/kg FA supplementation did not induce glucose metabolism disorder in male offspring. As an important methyl donor in vivo^9^, the effects of FA supplementation on the expression of DNMTs and insulin-related gene mRNA in the livers of mice need further study. In this study, when pregnant mice were exposed to LPS in the late stage of pregnancy, the mRNA level of *dnmt3a* was decreased and *pparγ* and *igf2* were increased in male offspring of pregnant mice supplemented with FA at 5 mg/kg compared with levels in male offspring of pregnant mice supplemented with folic acid at 2 mg/kg and 40 mg/kg. Moreover, the mRNA level of *dnmt3b* was increased and *peg3* was increased in male offspring of pregnant mice supplemented with folic acid at 5 mg/kg. Therefore, we hypothesized that appropriate FA supplementation can reduce the mRNA level of *dnmt3a*, decrease the methylation level of *pparγ* and *igf2*, and promote the mRNA levels of *pparγ* and *igf2*. Furthermore, it can promote the mRNA expression of *dnmt3b*, increase the methylation level of *peg3*, and inhibit the mRNA expression of *peg3*, thus improving glucose metabolism. Although the catalytic region of DNMTs is highly conserved, some amino acid residues are different, and methyl binding sites are different, which also explains why different DNA regions are methylated^28,29^.

PPAR*γ* is a ligand-activated transcription factor that belongs to the nuclear receptor family of peroxisome proliferation-activated receptors and plays an important role in the regulation of lipids, glucose homeostasis, inflammatory pathways and other processes. Mutation of the *pparγ* gene is associated with insulin resistance and T2D^30–32^. Van Otterdijk S D^33^ noted that *pparγ* DNA methylation levels were increased in T2D patients^30,34,35^. Clinically, T2D is treated with thiazolidinediones as insulin sensitizers, such as rosiglitazone^30,34,35^. These drugs are partial or complete agonists of PPARγ, which can increase insulin-stimulated glucose utilization and reduce hepatic glucose output to reduce blood glucose^30,34,35^. PPARγ is a key regulator of insulin sensitivity and can improve T2D through various mechanisms, such as increasing serum adiponectin levels, inhibiting inflammation, reducing mitochondrial reactive oxygen species production and promoting mitochondrial biosynthesis^34^. Mitochondrial dysfunction increases the production of reactive oxygen species, phosphorylates insulin receptor substrates, and further inhibits PI3K, AKT and other downstream insulin signalling pathways, reducing glucose production and insulin secretion^34^. In this study, the *pparγ* mRNA level of offspring in the FA5-LPS group was higher than that in the FA2-LPS and FA40-LPS groups.

IGF2 is critical for cell growth, survival, and metabolism^36^; is secreted with insulin; and is considered to be a physiological amplifier of insulin secretion^37^. IGF2 is associated with foetal growth and development and T2D^38^. Nica A C^39^ noted that the three most richly transcribed genes in the islets of nondiabetic people are *ins* (38%), *ins-igf2* (10%) and *igf2* (2%). IGF2 releases signals in an autocrine manner, modulates pancreatic β-cell function and increases insulin secretion under glucose stimulation to cope with metabolic stress and other adverse conditions^40,41^. IGF2 can bind to insulin receptor/IGF1 heterozygous receptor, recruit insulin receptor substrate protein, and activate the PI3K/AKT signalling pathway^36,42^. In this study, the *igf2* mRNA level in the offspring of the FA5-LPS group was higher than that of the FA2-LPS and FA40-LPS groups.

PEG3 is a zinc finger transcription factor that regulates cell growth, proliferation, and apoptosis^43^. Sojoodi^43^ showed that PEG3 inhibits the islet β-cell cycle, and reduced PEG3 expression leads to an increase in the number of circulating pancreatic β cells. Pancreatic β cells proliferate in response to increased metabolic demands, such as pregnancy, and insufficient β cells can lead to insulin deficiency and diabetes^44^. In this study, the *peg3* mRNA level in the offspring of the FA5-LPS group was lower than that of the FA2-LPS and FA40-LPS groups.

This study suggested that supplementation with 5 mg/kg FA seems to be an appropriate dose to ameliorate glucose metabolism disorder in male offspring induced by LPS exposure during pregnancy. FA is a universal methyl donor for all biological methylation reactions, participating in cell growth, proliferation, DNA synthesis and methylation^9,10^, and its deficiency can cause uracil misincorporation and DNA double strand breaks^45^. DNA methylation is closely linked to development, ageing and cancer^11^. Dietary deficiencies in FA are known to cause developmental defects, impair cognitive function, or block normal blood production^10^. Pfeiffer^46^ and Palchetti^47^ showed that the total FA concentration is closely associated with unmetabolized FA (UMFA). FA supplementation of 200 μg daily may result in the production of UMFA in blood and cord blood. UMFA refers to the FA that accumulates in biological fluids, presumably because the catalytic capacity of dihydrofolate reductase has been saturated^48,49^. Several reports have shown that higher FA/UMFA levels are associated with an increased risk of various disease conditions, including cancer, cardiovascular disease, diabetes and metabolic syndrome, unilateral retinoblastoma and obesity in offspring, other adverse birth outcomes, and autism^49–53^.

In conclusion, when pregnant mice were exposed to LPS in the late stage of pregnancy, FA supplementation of 5 mg/kg was more helpful in stabilizing the glucose metabolism disorder of their offspring than was FA supplementation of 2 mg/kg and 40 mg/kg.

## Supplementary Information

Supplementary Information.

## Data Availability

Data described in the current study are available from the corresponding author on reasonable request.

## References

[1] Barker DJ (2007). The origins of the developmental origins theory. J. Intern. Med..

[2] Hoffman DJ, Powell TL, Barrett ES, Hardy DB (2021). Developmental origins of metabolic diseases. Physiol. Rev..

[3] Simeoni U, Armengaud JB, Siddeek B, Tolsa JF (2018). Perinatal origins of adult disease. Neonatology.

[4] Vaiserman A, Lushchak O (2019). Developmental origins of type 2 diabetes: Focus on epigenetics. Ageing Res. Rev..

[5] Classification and Diagnosis of Diabetes: Standards of Medical Care in Diabetes-2020. *Diabetes Care* **43**: S14-s3110.2337/dc20-S002 10.2337/dc20-S002 31862745

[6] Fu L (2019). Oral cholecalciferol supplementation alleviates lipopolysaccharide-induced preterm delivery partially through regulating placental steroid hormones and prostaglandins in mice. Int. Immunopharmacol..

[7] Wolfson ML (2015). Lipopolysaccharide-induced murine embryonic resorption involves changes in endocannabinoid profiling and alters progesterone secretion and inflammatory response by a CB1-mediated fashion. Mol. Cell Endocrinol..

[8] Zhao M (2018). Maternal lipopolysaccharide exposure results in glucose metabolism disorders and sex hormone imbalance in male offspring. Mol. Cell Endocrinol..

[9] Friso S, Udali S, De Santis D, Choi SW (2017). One-carbon metabolism and epigenetics. Mol. Aspects Med..

[10] Lyon P, Strippoli V, Fang B, Cimmino L (2020). B vitamins and one-carbon metabolism: Implications in human health and disease. Nutrients.

[11] Wang M, Ngo V, Wang W (2021). Deciphering the genetic code of DNA methylation. Brief Bioinform..

[12] Ling C, Rönn T (2019). Epigenetics in human obesity and type 2 diabetes. Cell Metab..

[13] Ducker GS, Rabinowitz JD (2017). One-carbon metabolism in health and disease. Cell Metab..

[14] Liu HY, Liu SM, Zhang YZ (2020). Maternal folic acid supplementation mediates offspring health via DNA methylation. Reprod. Sci..

[15] Nasir A (2020). Nutrigenomics: Epigenetics and cancer prevention: A comprehensive review. Crit. Rev. Food Sci. Nutr..

[16] Caffrey A, McNulty H, Irwin RE, Walsh CP, Pentieva K (2019). Maternal folate nutrition and offspring health: Evidence and current controversies. Proc. Nutr. Soc..

[17] Bibbins-Domingo K (2017). Folic acid supplementation for the prevention of neural tube defects: US preventive services task force recommendation statement. JAMA.

[18] Field MS, Stover PJ (2018). Safety of folic acid. Ann. N. Y. Acad. Sci..

[19] Reeves PG (1997). Components of the AIN-93 diets as improvements in the AIN-76A diet. J. Nutr..

[20] Huang Y (2014). Maternal high folic acid supplement promotes glucose intolerance and insulin resistance in male mouse offspring fed a high-fat diet. Int. J. Mol. Sci..

[21] MacArthur Clark JA, Sun D (2018). Guidelines for the ethical review of laboratory animal welfare People's Republic of China national standard GB/T 35892–2018. Animal Model Exp. Med..

[22] Lin EE, Scott-Solomon E, Kuruvilla R (2021). Peripheral innervation in the regulation of glucose homeostasis. Trends Neurosci..

[23] Lennicke C, Cochemé HM (2021). Redox regulation of the insulin signalling pathway. Redox Biol..

[24] James DE, Stöckli J, Birnbaum MJ (2021). The aetiology and molecular landscape of insulin resistance. Nat. Rev. Mol. Cell Biol..

[25] Krishnaveni GV, Veena SR, Karat SC, Yajnik CS, Fall CH (2014). Association between maternal folate concentrations during pregnancy and insulin resistance in Indian children. Diabetologia.

[26] Xie K (2018). High folate intake contributes to the risk of large for gestational age birth and obesity in male offspring. J. Cell Physiol..

[27] Zetstra-van der Woude PA (2014). Maternal high-dose folic acid during pregnancy and asthma medication in the offspring. Pharmacoepidemiol. Drug Saf..

[28] Takeshima H (2006). Distinct DNA methylation activity of Dnmt3a and Dnmt3b towards naked and nucleosomal DNA. J. Biochem..

[29] Castillo-Aguilera O, Depreux P, Halby L, Arimondo PB, Goossens L (2017). DNA methylation targeting: The DNMT/HMT crosstalk challenge. Biomolecules.

[30] Cataldi S, Costa V, Ciccodicola A, Aprile M (2021). PPARγ and diabetes: Beyond the genome and towards personalized medicine. Curr. Diab. Rep..

[31] Yang Y, Chan L (2016). Monogenic diabetes: What It teaches us on the common forms of type 1 and type 2 diabetes. Endocr. Rev..

[32] Mirza AZ, Althagafi II, Shamshad H (2019). Role of PPAR receptor in different diseases and their ligands: Physiological importance and clinical implications. Eur. J. Med. Chem..

[33] van Otterdijk SD, Binder AM, Szarc Vel Szic K, Schwald J, Michels KB (2017). DNA methylation of candidate genes in peripheral blood from patients with type 2 diabetes or the metabolic syndrome. PLoS One.

[34] Wang S, Dougherty EJ, Danner RL (2016). PPARγ signaling and emerging opportunities for improved therapeutics. Pharmacol. Res..

[35] Cheng HS (2019). Exploration and development of PPAR modulators in health and disease: an update of clinical evidence. Int. J. Mol. Sci..

[36] Neirijnck Y, Papaioannou MD, Nef S (2019). The insulin/IGF system in mammalian sexual development and reproduction. Int. J. Mol. Sci..

[37] Livingstone C, Borai A (2014). Insulin-like growth factor-II: Its role in metabolic and endocrine disease. Clin. Endocrinol. (Oxf).

[38] Beaumont RN, Horikoshi M, McCarthy MI, Freathy RM (2017). How can genetic studies help us to understand links between birth weight and type 2 diabetes?. Curr. Diab. Rep..

[39] Nica AC (2013). Cell-type, allelic, and genetic signatures in the human pancreatic beta cell transcriptome. Genome Res..

[40] Arous C (2020). Integrin and autocrine IGF2 pathways control fasting insulin secretion in β-cells. J. Biol. Chem..

[41] Modi H (2015). Autocrine action of IGF2 regulates adult β-cell mass and function. Diabetes.

[42] Belfiore A (2017). Insulin receptor isoforms in physiology and disease: An updated view. Endocr. Rev..

[43] Sojoodi M (2016). The zinc finger transcription factor PW1/PEG3 restrains murine beta cell cycling. Diabetologia.

[44] Rieck S, Kaestner KH (2010). Expansion of beta-cell mass in response to pregnancy. Trends Endocrinol. Metab..

[45] Courtemanche C (2004). Folate deficiency and ionizing radiation cause DNA breaks in primary human lymphocytes: A comparison. Faseb. J..

[46] Pfeiffer CM (2015). Unmetabolized folic acid is detected in nearly all serum samples from US children, adolescents, and adults. J. Nutr..

[47] Palchetti CZ (2017). Association between serum unmetabolized folic acid concentrations and folic acid from fortified foods. J. Am. Coll. Nutr..

[48] Obeid R, Kasoha M, Kirsch SH, Munz W, Herrmann W (2010). Concentrations of unmetabolized folic acid and primary folate forms in pregnant women at delivery and in umbilical cord blood. Am. J. Clin. Nutr..

[49] Maruvada P (2020). Knowledge gaps in understanding the metabolic and clinical effects of excess folates/folic acid: A summary, and perspectives, from an NIH workshop. Am. J. Clin. Nutr..

[50] Selhub J, Rosenberg IH (2016). Excessive folic acid intake and relation to adverse health outcome. Biochimie.

[51] Morakinyo AO, Samuel TA, Awobajo FO, Oludare GO, Mofolorunso A (2019). High-dose perinatal folic-acid supplementation alters insulin sensitivity in sprague-dawley rats and diminishes the expression of adiponectin. J. Diet. Suppl..

[52] Li Z (2018). Folate and vitamin B12 status is associated with insulin resistance and metabolic syndrome in morbid obesity. Clin. Nutr..

[53] Tang JS (2022). MR1-dependence of unmetabolized folic acid side-effects. Front. Immunol..

