# Supplementary material for: Effects of folic acid supplementation in pregnant mice on glucose metabolism disorders in male offspring induced by lipopolysaccharide exposure during pregnancy
Source: Sci Rep. 2023 May 17;13:7984. doi: 10.1038/s41598-023-31690-w (PMC10192134; doi:10.1038/s41598-023-31690-w)

Fig 6A. GAPDH

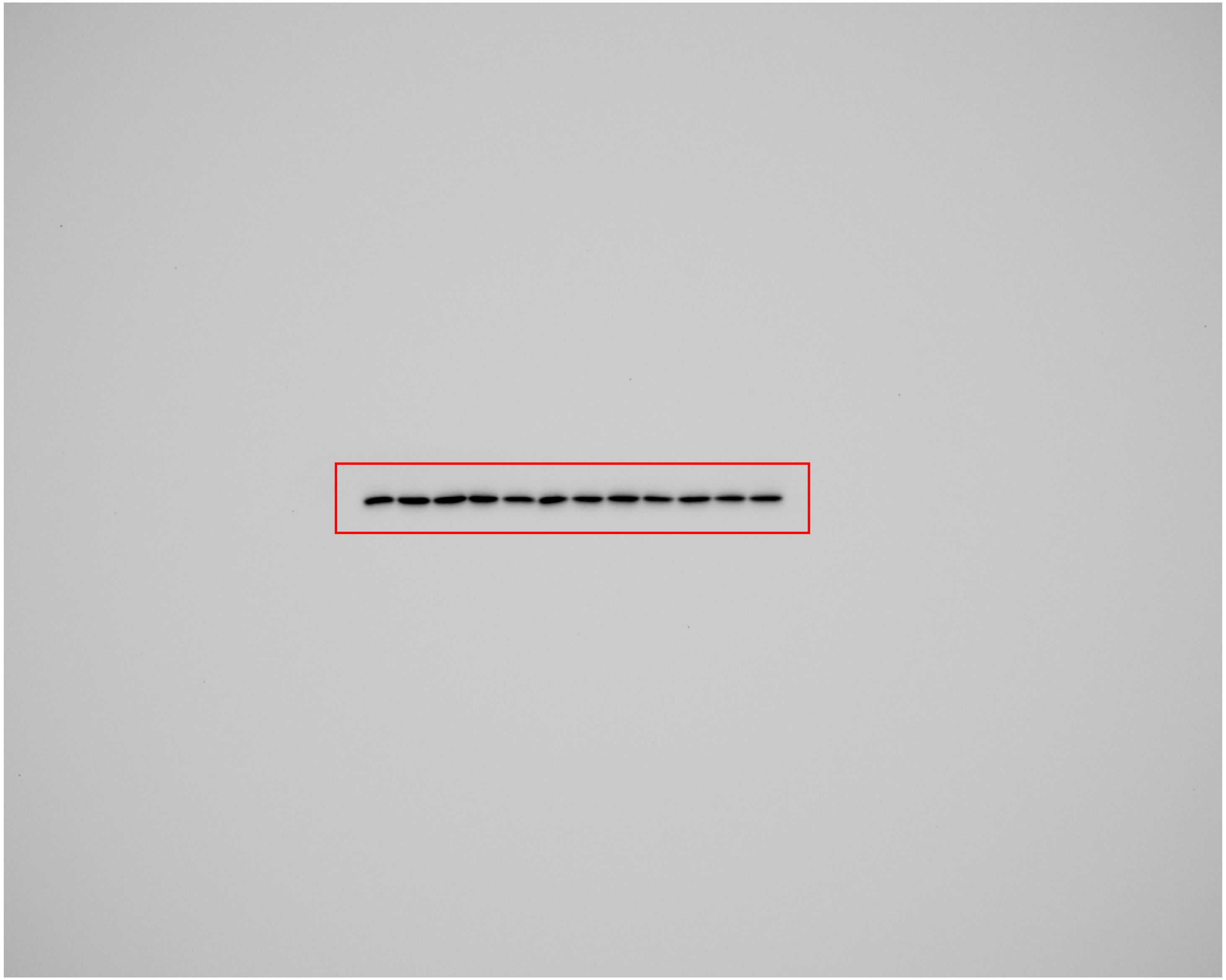

Fig 6A. p-PI3K

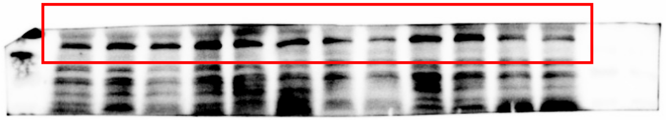

Fig 6A. PI3K

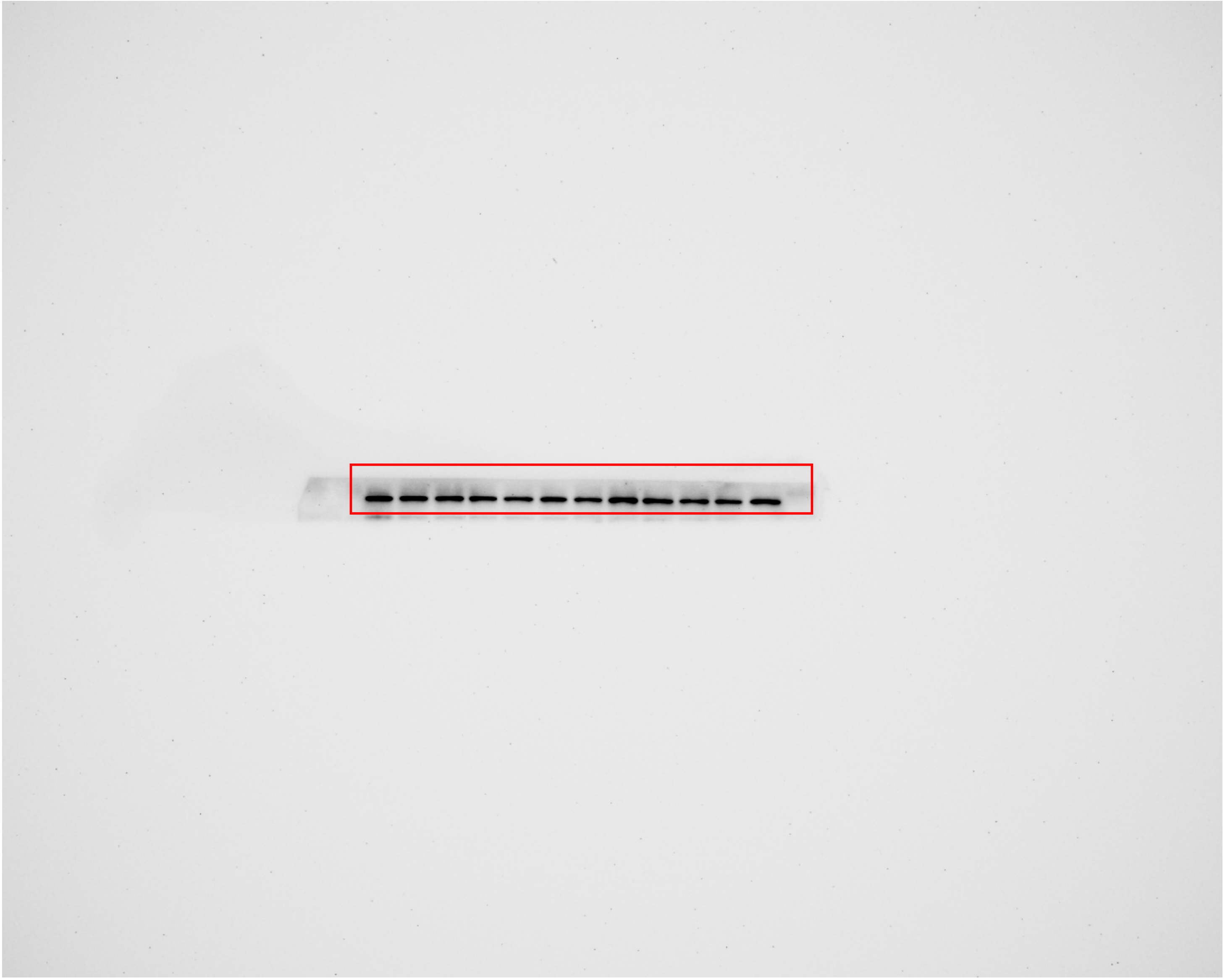

Fig 6B. GAPDH

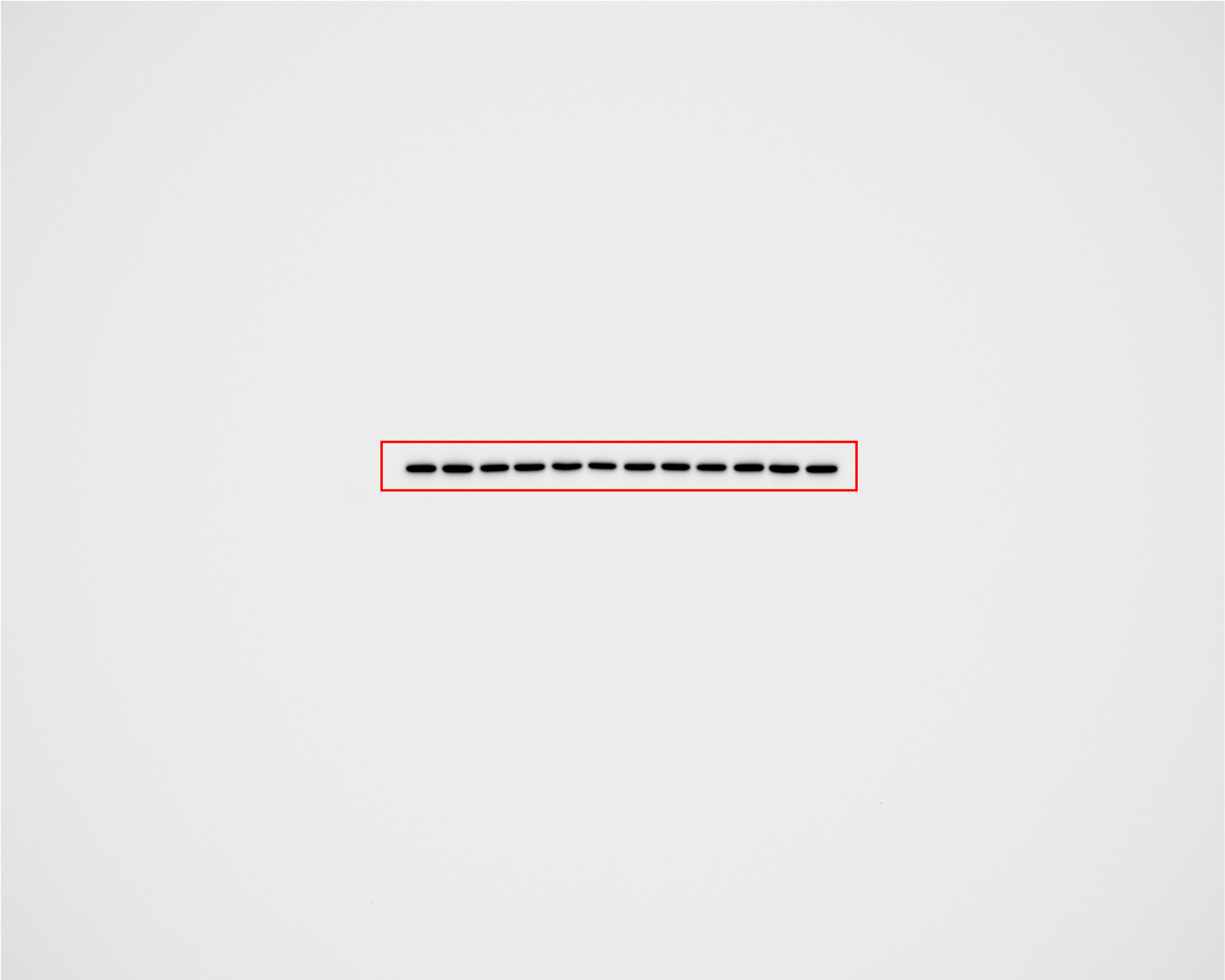

Fig 6B. p-AKT

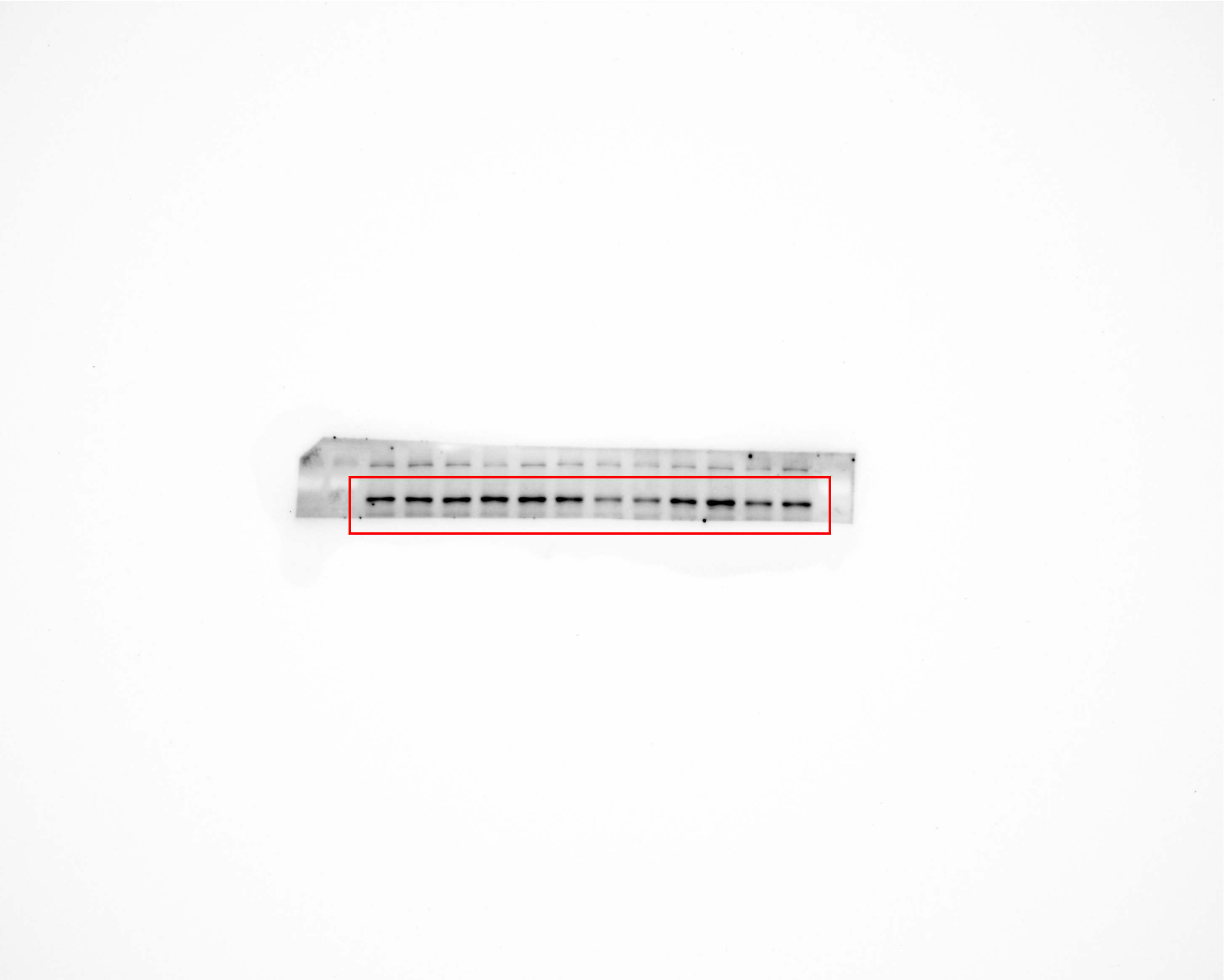

Fig 6B. AKT

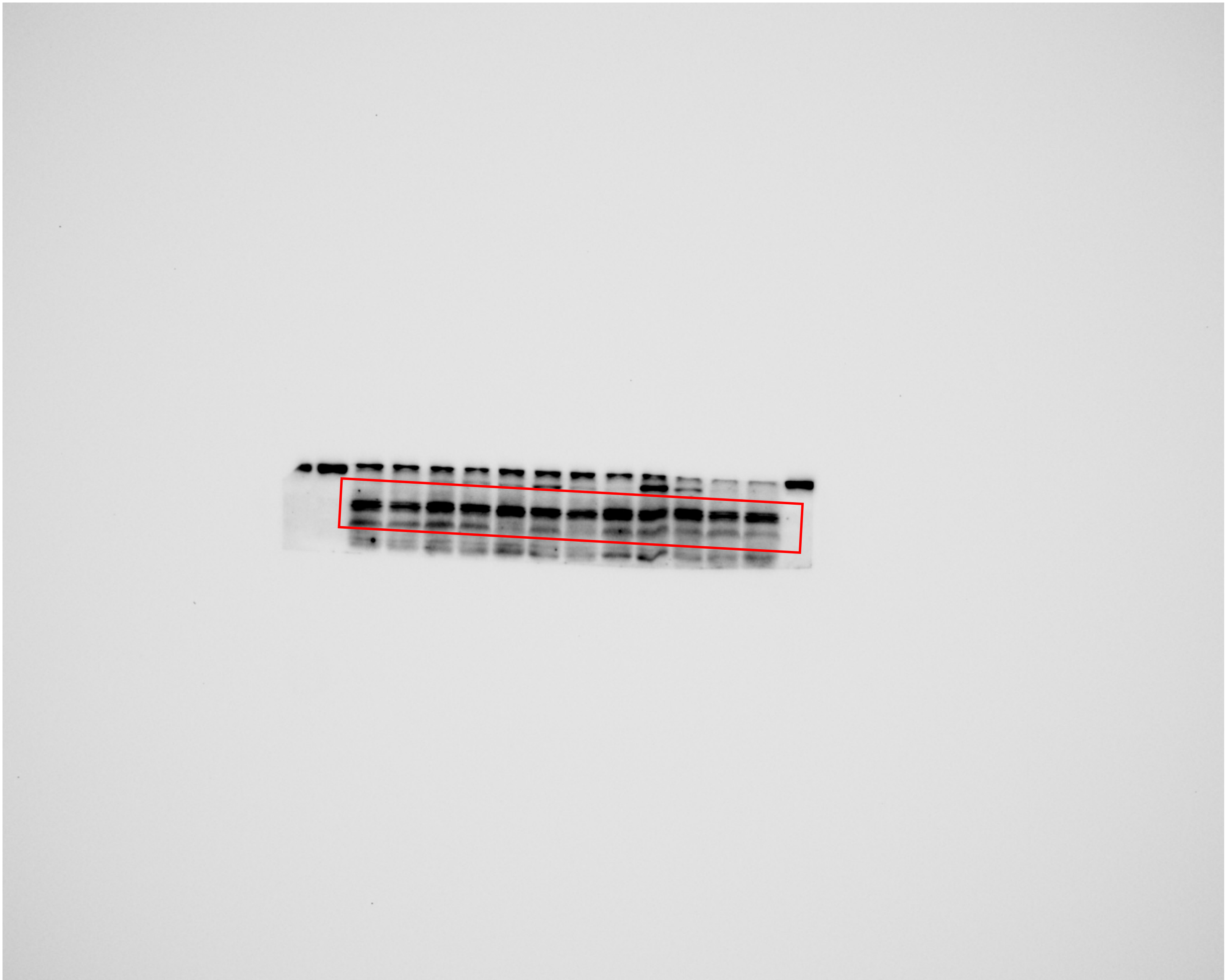

Fig 6C. GAPDH

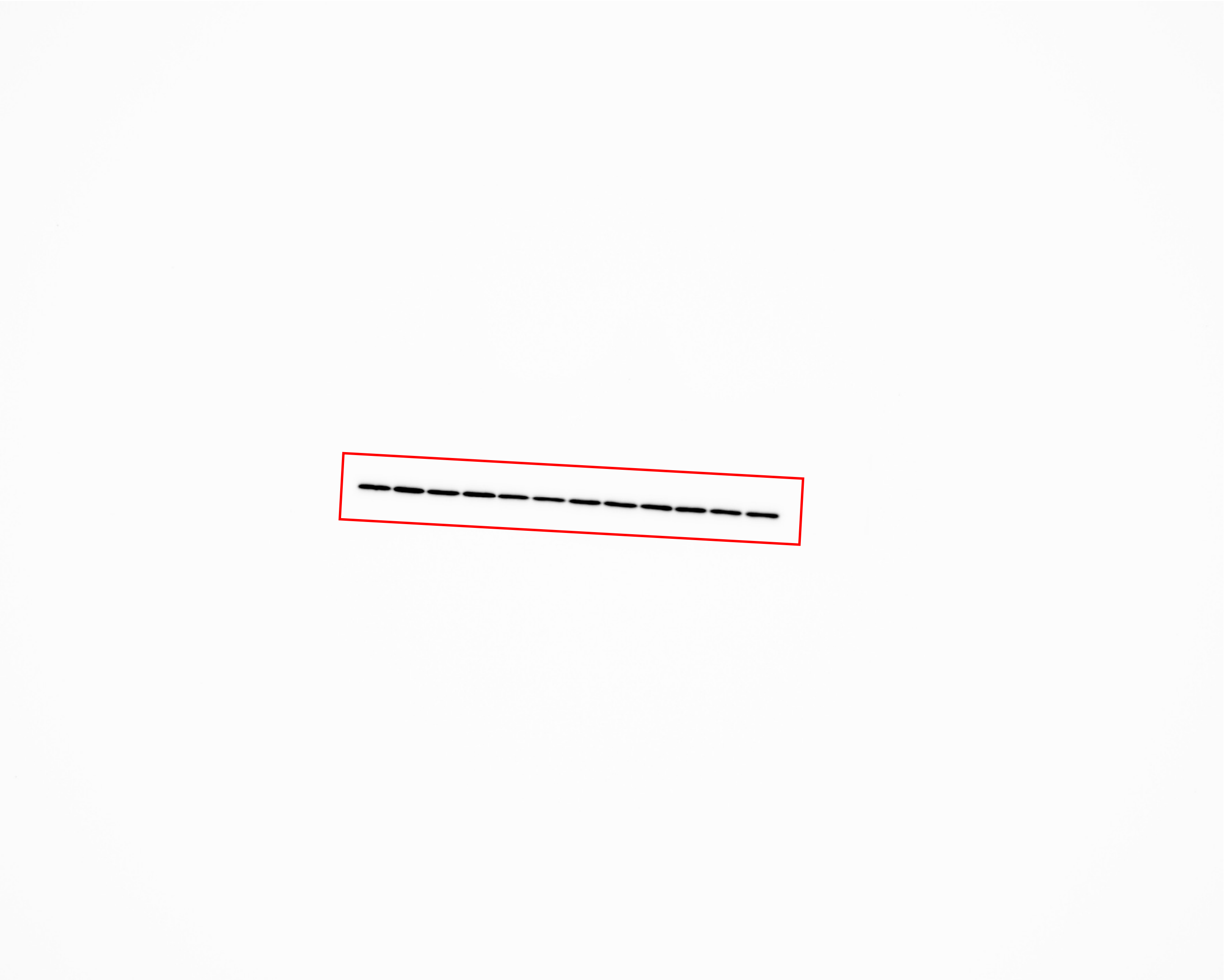

Fig 6C. p-GSK3β

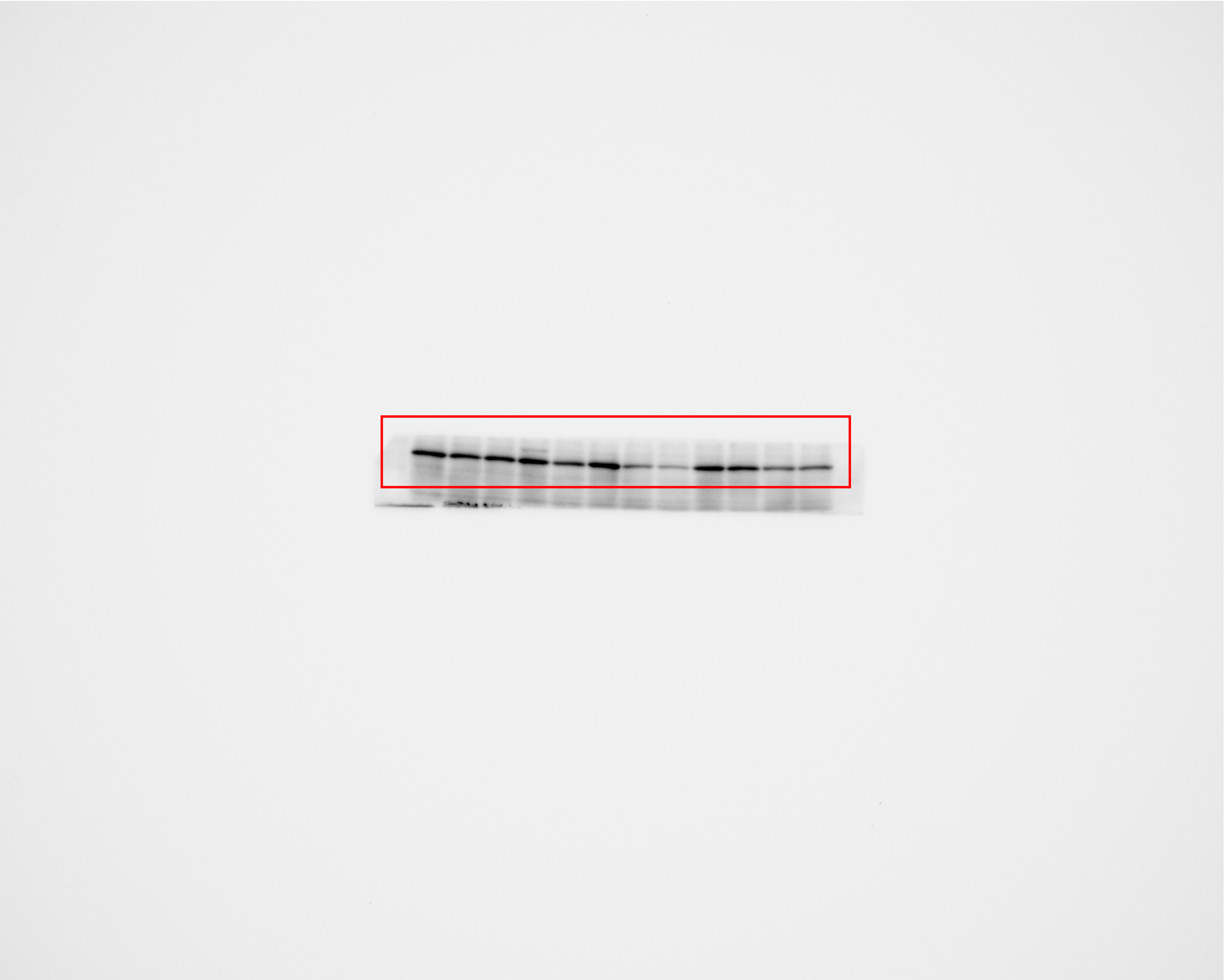

Fig 6C. GSK3β

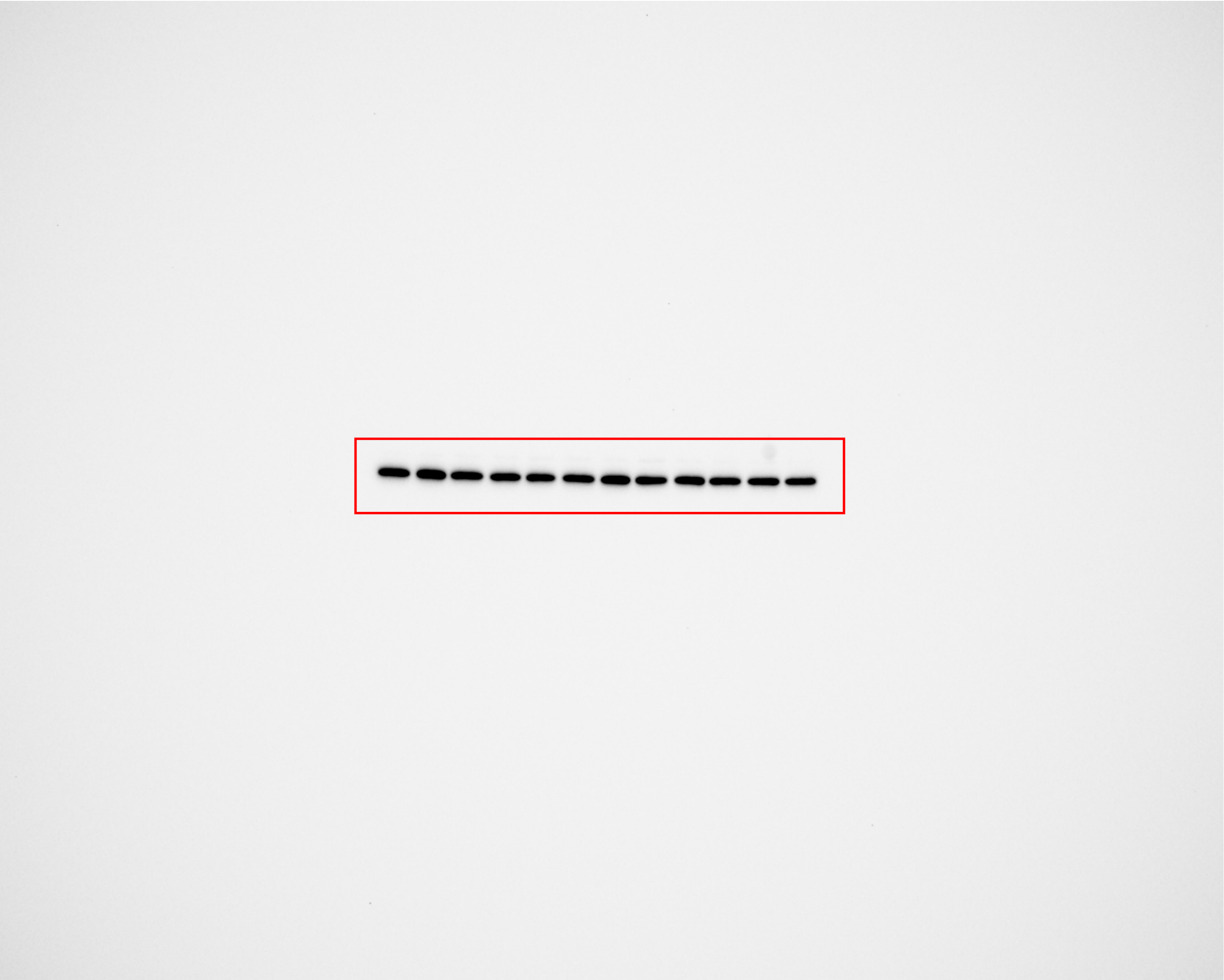

Supplement: Supplementary file 1 — Supplementary Information. [file 41598_2023_31690_MOESM1_ESM.pdf]
